# The future of suitable habitats of an endangered Neotropical grassland bird: A path to extinction?

**DOI:** 10.1002/ece3.9802

**Published:** 2023-02-14

**Authors:** Ricardo C. Meireles, Leonardo E. Lopes, Gustavo R. Brito, Ricardo Solar

**Affiliations:** ^1^ Pós‐graduação em Ecologia, Conservação e Manejo da Vida Silvestre Universidade Federal de Minas Gerais Belo Horizonte Brazil; ^2^ Laboratório de Biologia Animal, Instituto de Ciências Biológicas e da Saúde Universidade Federal de Viçosa Florestal Brazil; ^3^ Pós‐graduação em Biociências Universidade Estadual Paulista Assis Brazil; ^4^ Departamento de Genética, Ecologia e Evolução, Instituto de Ciências Biológicas Universidade Federal de Minas Gerais Belo Horizonte Brazil

**Keywords:** Campo Miner, climate changes, ecological niche modeling, land use changes, threatened species

## Abstract

Global changes increasingly worry researchers and policymakers and may have irreversible impacts on Earth's biodiversity. Similar to other phytophysiognomies, natural grasslands suffer from the effects of land use changes and rising temperatures, threatening animal and plant communities. Birds, being very sensitive to these changes, are widely studied and fundamental to understand the dynamics of ecosystems in relation to climate and land use changes. The Campo Miner *Geositta poeciloptera* is a grassland bird endemic to the Brazilian Cerrado and threatened with extinction that has been widely studied in recent years. We analyze the decrease in its extent of occurrence (EOO) and the effects of climate and land use change to understand the environmental suitability of the species in current and future scenarios. We used 5 common algorithms to produce ecological niche models. For future predictions, we use two general circulation models for two different greenhouse gas emission scenarios with different climate policies, an optimistic (ssp245) and a pessimistic (ssp585), plus two land use models focusing on increasing farmlands and reducing native grasslands. The current EOO represents ~45% of that presented by the IUCN EOO. The models generated for the present were satisfactory (TSS = 0.77 and ROC = 0.90) and showed high environmental suitability in areas where the species is currently found and low suitability where it is already extinct. All future scenarios have reduced suitable areas for the species, and the models of a greater increase in temperature and increase in farmlands and a greater decrease in grasslands were the worse. Our results reinforce the need to care about biome awareness disparity and the importance of actively preserving grassy‐shrub areas. Apparently, the state of Minas Gerais will be the only stronghold of the species in the coming years; however, the lack of protected areas that guarantee its survival needs attention.

## Graphical abstract

The Campo Miner is a grassland bird of the Brazilian Cerrado and threatened with extinction that has been suffering from the reduction of habitat. Here, we use ecological niche models to understand the environmental suitability of the species in the present and in the future. Our results show a marked reduction in climatically suitable areas for the species and reinforce the need to look carefully at the disparity in biome awareness and the importance of actively preserving grassy‐shrubland areas.

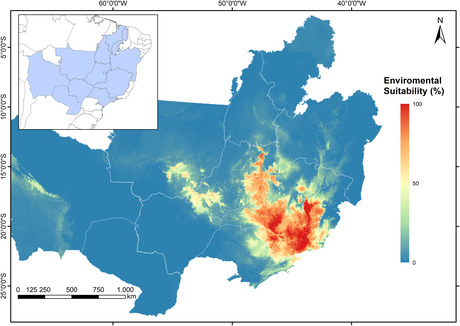

## 1 INTRODUCTION

Global changes have been a recurring concern among researchers and policy‐makers in recent years. According to the latest report of the Intergovernmental Panel on Climate Change, the world may experience an alarming increase in temperature, reaching or exceeding 1.5°C in the next two decades, leading to more dangerous extreme weather events and irreversible damage to the earth (IPCC, 2022). Effects such as ocean acidification, rising seas, rain, and drought extremes, among others, will intensify, causing, in addition to impacts on human survival, a great loss of biodiversity (IPCC, 2022) that, together with land use change, will be the main causes of species extinction by 2100 in virtually every terrestrial ecosystem of the planet (Sala et al., 2000).

For grassland ecosystems, climate projections show a substantial increase in temperature and, added to land exploitation and habitat loss, may further compromise the composition of native species (Gibson & Newman, 2019; Sala et al., 2000). For the Brazilian Cerrado, a biogeographic province with different types of grassland, the projections are not encouraging either. Hofmann et al. (2021) show that the increase in temperature will reduce the relative humidity by ~15%, making these grasslands increasingly drier and hotter, directly affecting local biodiversity. This finding becomes even more worrying since open areas are still poorly understood and with many erroneous restoration policies (Silveira et al., 2021). Additionally, the Cerrado is one of the biogeographic provinces with great richness of animal and plant species in the world, being also a biodiversity hotspot (Myers et al., 2000).

As a group that is very sensitive to global changes, birds are widely studied and are fundamental to understand the dynamics of ecosystems in relation to climate and land use changes (Borges & Loyola, 2020; Borges et al., 2019; Marini et al., 2009a; Moraes et al., 2021). Among the Cerrado grassland birds, the Campo Miner *Geositta poeciloptera* is a threatened species that since 2012 has been subject to a long‐term study that focused on its basic natural history (Lopes & Peixoto, 2018; Machado et al., 2017), reproductive ecology (Meireles et al., 2018, 2021), environmental endocrinology (Lopes et al., 2021), and movement ecology (Lopes et al., 2023). Endemic to the Cerrado and with its range almost restricted to Brazil (Lopes et al., 2023), the species inhabits open landscapes with sparse grass cover and exposed soil (Lopes & Peixoto, 2018; Machado et al., 2017) and is suffering a marked decrease in its area of occurrence over the years, largely due to land use changes (BirdLife International, 2022; Lopes et al., 2009; Silveira, 2009). Marini et al. (2009a) in their study with several endemic birds of the Brazilian Cerrado, including the Campo Miner, pointed out a drastic decrease in their area of the occurrence until 2100 due to climate changes. However, the study considered only few information about the natural history of the species that were available at that time, not identifying in detail the priority areas for its conservation.

In this study, we used occurrence records obtained from different sources of data, in addition to records collected in the field, to understand the past and current distribution of Campo Miner and to discuss the possible causes of the disappearance of the species in certain regions. Furthermore, using ecological niche modeling, we investigate the impacts of climate and land use changes on suitable habitats for the species in the present and future. With this, we want to understand why its range is being reduced and where the most favorable areas for its occurrence and conservation are located. For this, we evaluated possible changes in the size and location of suitable areas in different scenarios of greenhouse gas emission policies and changes in land use. Due to the lack of prospects for a return to preindustrial emission levels (IPCC, 2022) and effective enforcement policies regarding land use, we expect to find a decrease in suitable areas in all scenarios. However, we expect that the scenario with the larger increase in emissions (and consequently a greater increase in terrestrial temperature) and greater loss of natural grasslands will result in a greater loss of suitable areas.

With this paper, together with the knowledge acquired during the last 10 years of studies with Campo Miners, we expect to better understand the possible causes of its local extinction and draw up plans for its conservation, as well as understand the ecology of grassland ecosystems in the face of climate change and its importance for biodiversity.

## 2 METHODS

### 2.1 Study species and area

The Campo Miner is a threatened grassland terrestrial passerine (Machado et al., 2017; Ridgely & Tudor, 2009) classified as Vulnerable in Brazil (MMA, 2022) and globally (BirdLife International, 2022). In the state of São Paulo, the species is considered regionally extinct (Alesp, 2018). For these reasons, and also for the low protection of the species in reserves (Marini et al., 2009b), it was included in the National Action Plan (PAN) for the Conservation of Cerrado Birds (ICMBio, 2021a).

The Campo Miner inhabits the more open grasslands of the Cerrado savannas (Lopes & Peixoto, 2018; Machado et al., 2017; Ridgely & Tudor, 2009), a Brazilian biogeographic province that suffers from anthropogenic impacts (ICMBio, 2021b) and climate and land use changes, with severe impacts upon the species' conservation (Hofmann et al., 2021; Marini et al., 2009a). There are also scarce records of the species for the Cerrados of Bolivia and Paraguay, from where it is known from historical specimens (del Castillo et al., 2005; Herzog et al., 2016). The Campo Miner is a habitat specialist, living in very open grasslands growing on shallow soils, which show patches of exposed soil and that suffer a high incidence of erosion processes, which expose the soil banks where the species excavate the burrows where it nest (Lopes & Peixoto, 2018; Meireles et al., 2018).

**FIGURE 1:**
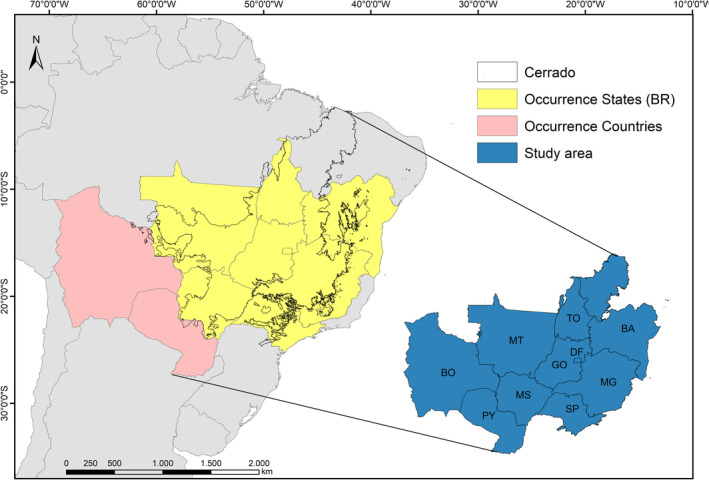
Study area encompassing the Cerrado biogeographic province, the states of occurrence of the Campo Miner in Brazil (Occurrence States—BR), and other countries of occurrence (Occurrence Countries). States: BA = Bahia, DF = Federal District, GO = Goiás, MG = Minas Gerais, MS = Mato Grosso do Sul, MT = Mato Grosso, SP = São Paulo, TO = Tocantins. Countries: BO = Bolivia, PY = Paraguay.

Due to its distribution, we used the entire boundary of the Cerrado, covering the three countries of occurrence (Brazil, Bolivia, and Paraguay), as the study area, also considering all Brazilian states where there are records of occurrence of the species (see Lopes et al., 2023; Figure 1).

### 2.2 Species record data

To understand the distribution of the Campo Miner, we used the occurrence records previously collected by Lopes et al. (2023). These data are from a literature review conducted in two search engines (https://scholar.google.com and www.biodiversitylibrary.org) using the following keywords: Campo Miner, *Geositta poeciloptera*, and *Geobates poecilopterus* (a previous name of the species). This dataset also includes data from online sound archives (http://macaulaylibrary.org, www2.ib.unicamp.br/fnjv), citizen science platforms (www.xeno‐canto.org, www.wikiaves.com.br, and http://ebird.org), museum databases (www.vertnet.org, http://splink.cria.org.br) and general biodiversity databases (www.gbif.org). A total of 15 Brazilian and overseas ornithological collections were also personally visited (see Lopes et al., 2023).

Due to the authors' previous field experience and knowledge about the species' natural history, habitat preference, and strict nesting requirements (Machado et al., 2017; Meireles et al., 2018, 2021), we excluded from the analysis records obtained in sites that do not harbor suitable habitats for it.

We also obtained occurrences records from other ornithologists coming into direct contact with them, in addition to records made in the field by the authors themselves (see Lopes et al., 2021, 2023; Lopes & Peixoto, 2018; Machado et al., 2017; Meireles et al., 2018, 2021).

For all records found, we organized the database by location, date (<1950, 1950–1990, and >1990), and geographic coordinates. The choice of cut‐off dates was based on the study by Lopes et al. (2023). For those records to which it was not possible to accurately identify their exact geographic location, we adopted the municipal seat as a reference. After constructing our database of occurrence data, we removed all duplicate points.

### 2.3 Extent of occurrence (EOO)

We used the occurrence records obtained above from 1990 to 2021 to estimate the current Extent of Occurrence (EOO) of Campo Miners. According to the IUCN (2012) definition, the EOO is “the area contained within the shortest continuous imaginary boundary that can be drawn to encompass all the known, inferred or projected sites of present occurrence of a taxon, excluding cases of vagrancy.” For this, we build a convex hull polygon in the ConR package (Dauby, 2020). We also performed a comparison between the EOO generated by us and the EOO presented on the IUCN website and available by BirdLife International and Handbook of the Birds of the World (2021).

### 2.4 Predictions

#### 2.4.1 Variables

To build the variables database, we obtained bioclimatic data from South America from the WorldClim v2.1 database (Fick & Hijmans, 2017). We performed a test of Variance Inflation Factor (VIF) analysis using the usdm package (Naimi et al., 2014) to minimize multicollinearity in the data (the default limit of 10 was used). We also obtained the land use models from data provided by Li et al. (2017). All data were in 2.5‐min resolution, Datum WSG84, and were cropped according to the study area (Figure 1). To minimize spatial autocorrelation and clustering, we create a 5 km buffer around each presence record using the spThin package (Aiello‐Lammens et al., 2015).

#### 2.4.2 Present prediction

To predict the current environmental areas suitable for Campo Miner, we only used occurrence records with exact coordinates and after 1990 (*n* = 47) since the records prior to this date are located in anthropized areas or even where the species is considered extinct (Bressan et al., 2009; Lopes et al., 2009), as well as the historical records (<1950) that were also not considered because the location of the record may not correspond to the current environmental reality of the species. We built all models using the biomod2 package (Thuiller et al., 2021) and five algorithms of ecological niche modeling (ENM; Table 1) with 10‐fold cross‐validation. To train and test the models, 75% and 25% of the data were kept during each run, respectively. Model performance was determined by a threshold >0.7 and assessed based on the average True Skill Statistic—TSS—(Allouche et al., 2006) and receiver‐operating characteristic—ROC—curve (Fielding & Bell, 1997).

**TABLE 1:** Ecological Niche modeling selected algorithms for the Campo Miner distribution (adapted from Raes & Aguirre‐Gutiérrez, 2018).

Group	Algorithm	Description	“Absence” data	Number of “absence” records	Generation	References
I	SRE	Surface range envelope	Background data	10,000	Random across the environment	Nix (1986); Busby (1991)
I	MAXENT	Maximum entropy	Background data	10,000	Random across the environment	Phillips et al. (2006)
II	BRTs	Boosted regression trees	Pseudo‐absence	100	0.5‐degree wide buffer around presence point	Elith et al. (2008)
II	RFs	Random forests	Pseudo‐absence	100	0.5‐degree wide buffer around presence point	Breiman (2001)
II	GLMs	Generalized linear models	Pseudo‐absence	100	0.5‐degree wide buffer around presence point	McCullagh and Nelder (1989); Venables and Ripley (2002)

Given that the engines of selected algorithms are different, we followed Barbet‐Massin et al. (2012) recommendations to generate background and pseudo‐absence data. For that, we divided the algorithms into two groups according to the type of “absence” data they require, as shown in Table 1, being 100 pseudo‐absence points for BRT, RF, and GLM algorithms (weighting the “absences” based on the number of presences) and 10,000 background points for Maxent and SRE algorithms, in accordance to Phillips et al. (2006).

We selected the best models for ensembling based on the TSS value, maximizing sensitivity and specificity, generating five ensembles, one per algorithm (Liu et al., 2005). Afterwards, we used the weighted average of the binarized projections of the determined TSS limit, resulting in model agreement for each group, following the method used by Köhler et al. (2020). From the two ensemble model groups generated, we superimposed the maps to compute the average of the overlapping pixel values to generate a single map of the present prediction.

#### 2.4.3 Future predictions

For future predictions, we used two General Circulation Models (GCM) with good performances in simulating precipitation and air surface temperature for the Cerrado region (see Ortega et al., 2021): MPI‐ESM1‐2‐HR and MRI‐ESM2‐0. The scenarios modeled for the Campo Miner comprise the years 2041–2060 and 2061–2080. For both GCMs, we evaluated two different greenhouse gas emissions scenarios with different climate policies (Shared Socioeconomic Pathways—SSPs), one being an optimistic scenario—ssp245—with a slow decrease in greenhouse gas emissions limit warming around 2.5°C over the years and, a pessimistic scenario, with substantially higher greenhouse gas emissions—ssp585—increasing ~5°C until 2100 (Gidden et al., 2019; Riahi et al., 2017). All scenarios were based on the Coupled Model Intercomparison Project Phase 6 (CMIP6) by Eyring et al. (2016) and the data were also obtained from the WorldClim v2 database (Fick & Hijmans, 2017).

Land use models for the future were obtained from data provided by Li et al. (2017). We resorted to two different scenarios, one with a moderate increase in farmlands and a moderate decrease in grassland areas (A1B) and the other with a greater increase in farmlands and a greater decrease in grassland areas (A2). We then related each model to the respective climate scenario: (1) A1B to ssp245; (2) A2 to ssp585. Due to the lack of a period comprising the analyzed climate periods, we used land use change models of 2050 for the 2041–2060 climate period and 2100 for the 2061–2080 climate period. All analyses (spatial correlation, EOO, present, and future predictions) that used specific packages were performed in R (R Development Core Team, 2021).

#### 2.4.4 Present and future estimates of the environmentally suitable area

We used ArcGis 10.5 (ESRI, 2016) to calculate the loss and/or gain of suitable environmental areas for Campo Miners. From current occurrence records (>1990), we selected areas above 50% suitability. This choice is due to the fact that these occurrence records are mostly found in this area, which makes the probability of occurrence greater in more suitable areas. For this, we used the niche models generated for the present and future, vectorized the maps, and calculated their geometry (>50%) in km^2^.

## 3 RESULTS

**FIGURE 2:**
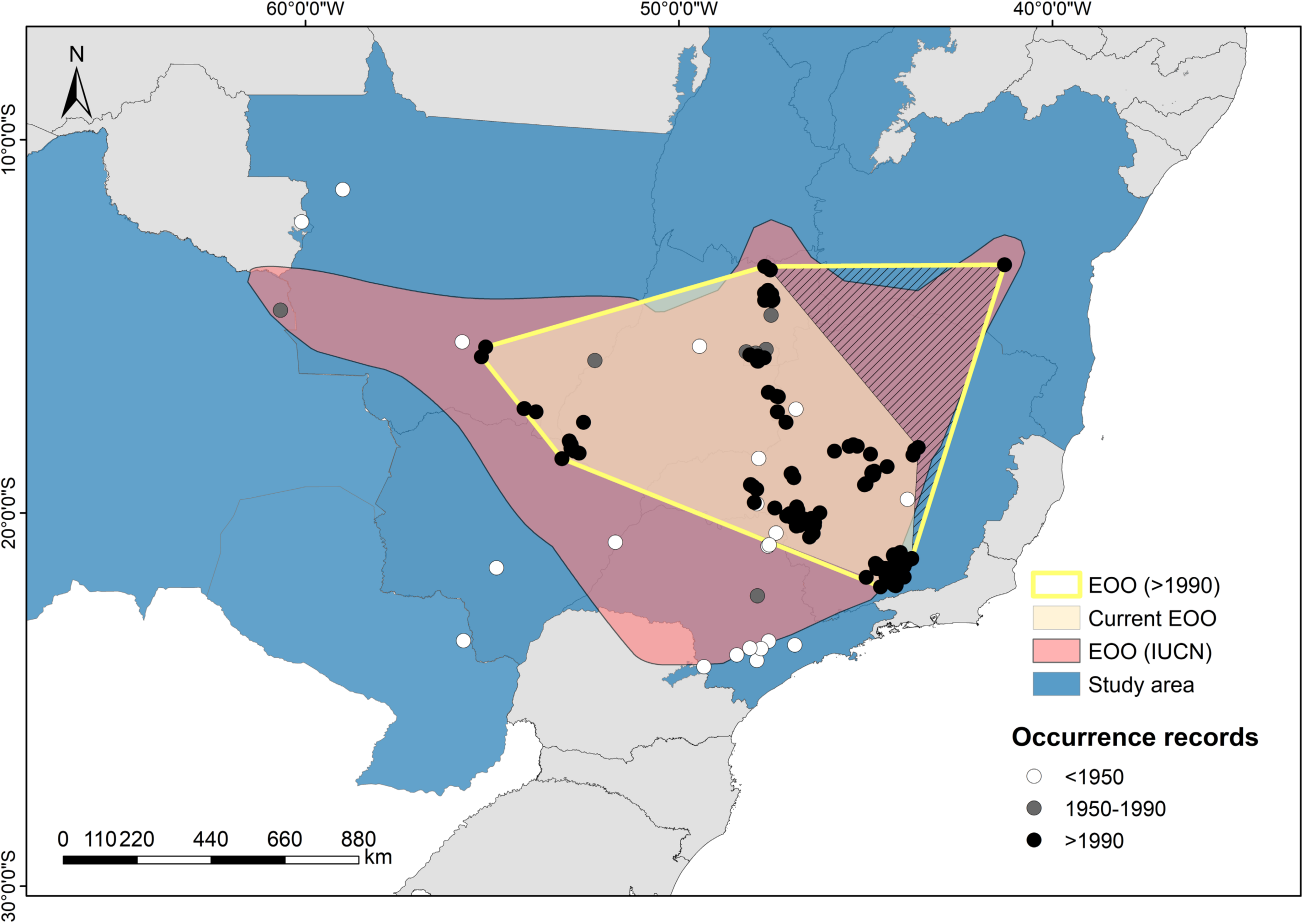
All occurrence records of Campo Miners and the comparison between the EOO based only on records >1990 (with or without) the occurrence Bahia state (hatched area), where the species is probably extinct and the EOO available on the IUCN website.

A total of 1110 occurrence records were found for the species in 67 municipalities in 6 Brazilian states and Bolivia and Paraguay. After filtering to remove duplicate points, we obtained about 264 records. The first records of the species were obtained in the state of São Paulo in 1819 in Ipanema (today Ipanema National Forest, municipality of Iperó) and along the road to the municipality of Sorocaba. Almost all records for the state of São Paulo were obtained before 1950, with only one record obtained between 1950 and 1990. Records for the northwest part of Mato Grosso, southwest and eastern part of Mato Grosso do Sul were also obtained before 1950. Most of the records, irrespective of the period considered, are from the Brazilian states of Goiás, Minas Gerais, and Distrito Federal. Records for Bolivia and Paraguay were obtained in the years 1989 and 1938, respectively (Figure 2).

### 3.1 Extent of occurrence

The current extent of occurrence with records over 1990 covers an area of 889,509 km^2^, ~39% smaller than that estimated by the IUCN (1,457,020 km^2^). Both estimates cover the states of Minas Gerais, Goiás, Mato Grosso, Mato Grosso do Sul, Bahia, Tocantins, and Distrito Federal, with a larger (IUCN EOO) or smaller (current EOO) area delimited for each state. In addition, the IUCN EOO also extends to the state of São Paulo and beyond Brazil, covering a small part of Bolivia (Figure 2).

### 3.2 Predictions

After verifying the multicollinearity of the 19 bioclimatic variables, only 7 were selected (Table 2). Annual mean temperature (BIO1) proved to be the main variable representing 64.5% of contribution to the occurrence of Campo Miners, followed by precipitation of the wettest quarter (BIO16) with 32.8% of contribution (Table 2).

**TABLE 2:** Selected bioclimatic variables to predict the present distribution of the Campo Miner.

Variables	Type	Description	Percent contribution (%)
BIO1	Cont.	Annual Mean Temperature	64.5
BIO2	Cont.	Mean Diurnal Range (Mean of monthly (max temp ‐ min temp))	7.2
BIO3	Cont.	Isothermality	10.2
BIO15	Cont.	Precipitation Seasonality	9.8
BIO16	Cont.	Precipitation of Wettest Quarter	32.8
BIO18	Cont.	Precipitation of Warmest Quarter	7.8
BIO19	Cont.	Precipitation of Coldest Quarter	14.0

Abbreviation: Cont., Continuous.

All 50 models generated from the 5 chosen algorithms (10 runs for each one) had good performances, presenting an average of the values of TSS = 0.77 and ROC = 0.90, higher than the 0.7 threshold previously determined.

**FIGURE 3:**
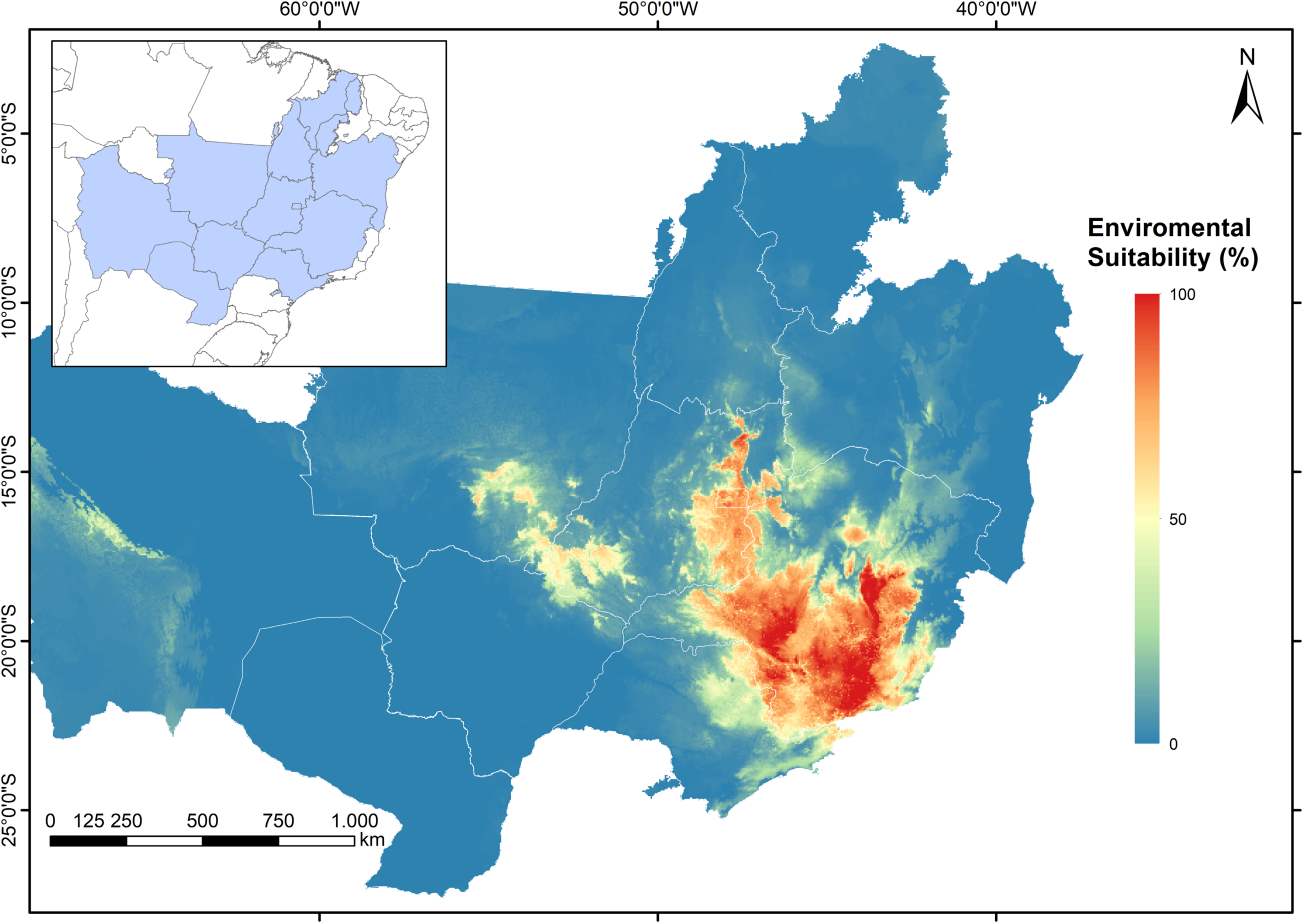
Ensemble model generated for the prediction of the currently suitable areas for the occurrence of Campo Miner.

The estimate of areas above 50% suitable for the occurrence of Campo Miners was ~348,293 km^2^ and covered, as expected, mainly the Cerrado (Figure 3) and all regions where the species was currently recorded (see Appendix S1: Figure 1), which shows a good performance of the models generated for the present.

**FIGURE 4:**
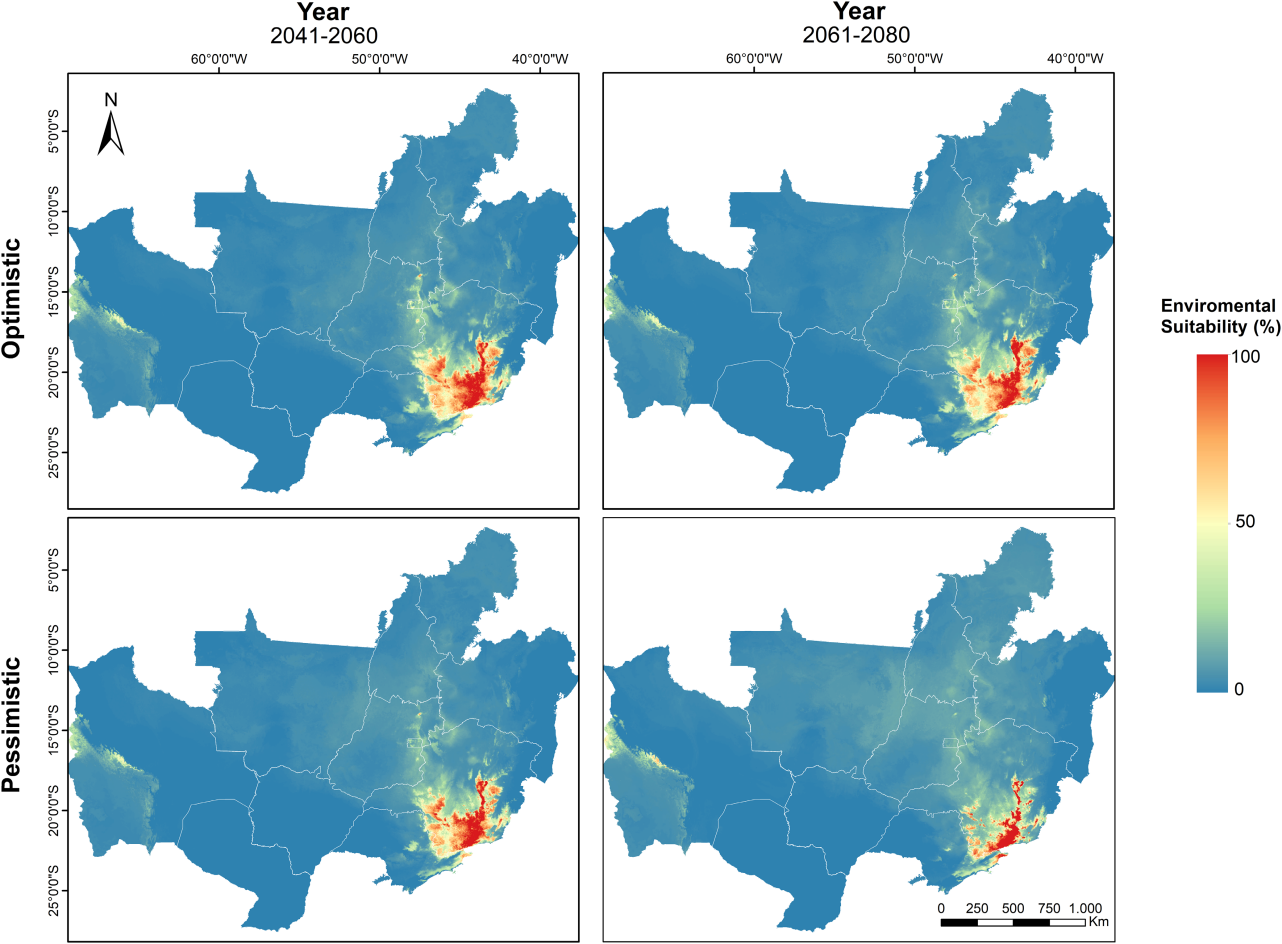
Future prediction of suitable areas for the occurrence of Campo Miners in two different scenarios (Optimistic—ssp245 and Pessimistic—ssp585). See all the scenarios in Appendix S1: Figure 2.

As with the current prediction, the maps generated by the two groups of algorithms were superimposed, generating a total of four projections. They were separated by period (2041–2060 and 2061–2080) and optimistic and pessimistic scenarios (Figure 4). All models show the reduction of suitable habitats for the occurrence of the Campo Miner (> 50%) in a period of approximately 50 years. The most pessimistic scenario with a substantially greater increase in greenhouse gas emissions, a greater increase in farmlands, and a decrease in native grasslands presented the greatest reduction in area, with its areas estimated at 79,426.58 km^2^ for 2041–2060 and 25,140.02 km^2^ for 2061–2080, which represents approximately 77–92%, respectively, of the area lost in relation to the current model. The optimistic scenario, even with stricter climate and land use policies, also showed a considerable reduction in areas >50% for the occurrence of Campo Miner, with estimates of 108,163.85 km^2^ for 2041–2060 and 87,858.19 km^2^ for 2061–2080, approximately 68–74%, respectively, area reduction compared with the present (Figure 4).

## 4 DISCUSSION

The current EOO of Campo Miners in Brazil has suffered a dramatic reduction in recent years, with several regions exhibiting no record after 1950. Although the IUCN EOO covers almost the entire area with records of Campo Miners in the last two centuries (including Bolivia) and excludes records from where the species is extinct (e.g., some areas of São Paulo, northwest Mato Grosso, and Paraguay), it is currently outdated, because other regions with historical records no longer harbor the species. Furthermore, the current EOO may be even smaller, as the single record of the species for Bahia was published by Parrini et al. (1999), who recorded the species close to the locality of Brejo, along the road from Ibicoara to Jussiape, on 9 December 1995 (Parrini pers. com.). Most of the grasslands that originally covered this region have been lost, mainly converted to temporary crops (Parrini pers. com., Google Earth images). We suspect that the species is extinct in Bahia and, therefore, if we do not consider this single occurrence for the state, the entire hatched area in Figure 2 would need to be excluded from the current EOO of Campo Miners. Therefore, the current EOO will be ~659,981.3 km^2^, about 55% smaller than the EOO proposed by the IUCN.

### 4.1 Present prediction

The fact that the variables Annual Mean Temperature (BIO1) and Precipitation of Wettest Quarter (BIO16) are the most important for the distribution of the species, with 64.5% and 32.8% of contribution, respectively, shows us how much its distribution occurs in places of similar climates. The Campo Miner is found mostly in the central and southeastern regions of Brazil, which have an Aw and Cwb climate according to the Köppen classification (Álvares et al., 2013) with an average annual temperature for the states of occurrence of approximately 23°C (Aw) and 18°C (Cwb). In addition, both climate types have dry winters and wet summers (Álvares et al., 2013) with the rainy season being preferred for reproduction, with a reproductive peak in late October to early November, but extending into December (Machado et al., 2017; Meireles et al., 2021), being the wettest quarter of the year.

Four regions that harbor important strictly protected areas for the species (ICMBio, 2011) showed high levels of environmental suitability in the present, namely: (1) Brasília National Park, Distrito Federal; (2) Chapada dos Veadeiros National Park, Goiás; (3) Emas National Park, Goiás; and (4) Serra da Canastra National Park, Minas Gerais. All of them harbor vast expanses of grasslands and can be of paramount importance for the conservation of the species (ICMBio, 2021b). However, in Brasília National Park the species seems already to be currently very rare (Braz, 2008, WikiAves data).

The areas with the lowest environmental suitability (<50%) are mostly in regions where the species has already been declared extinct, as follows:

1. São Paulo state: Although researchers still indicate the possibility of the species occurring in the state (Bressan et al., 2009), the Alesp (2018) decree and other studies point to its extinction (Motta‐Junior et al., 2008; Willis, 2004). The last record for the state was obtained in 1987.
2. Mato Grosso state: records for the northwest part of the state were obtained in the Chapada dos Parecis, a poorly studied region that was briefly visited by LEL in the 2010s. Despite still harboring vast areas of natural grasslands with apparently suitable habitats for the species, no Campo Miner was observed during this brief visit. For the well‐sampled Chapada dos Guimarães, the latest bird surveys did not find the species, and only century‐old museum records are known from there (Dornas, 2020; Lopes et al., 2009). In brief, all records available for these regions occurred before 1950.
3. Bahia state (Chapada Diamantina): As mentioned earlier, only one record of the species was made there in the 1990s (Parrini et al., 1999). As it is a highly touristic area and with a high flow of people (including birdwatchers), the lack of records in the last 30 years (see www.wikiaves.com.br) seems to confirm the hypothesis of extinction in the region.
4. Mato Grosso do Sul state: Only two records of the species for the Southwest region were found in 1938 on the Corralinho farm. There is still a record for the eastern region in the municipality of Três Lagoas in 1932 and there is no more information about the species in the region.
5. Bolivia: In Bolivia, this species is only known from the Huanchaca Plateau, where it was last recorded in 1989 (Bates et al., 1992; Herzog et al., 2016). Most of this region, however, is still undisturbed and well protected within the Noel Kempff National Park, and the possibility that small populations of this species still persist the region that cannot be disregarded.
6. Paraguay: The only record for the region was in 1938 (del Castillo et al., 2005); however, the anthropization of the region and the high loss of native flora in the Cerrado areas in the country (Velazco et al., 2019) add to the possible local extinction of the species.

The possible cause of the extinction of the species in these regions is attributed to the wiping of natural grasslands, replaced by exotic pastures and crops (Lopes et al., 2009; Silveira, 2009). The lack of proper fire management needs also to be considered, as the species is fire‐dependent, occupying recently burned areas (Lopes et al., 2023; Machado et al., 2017; Willis, 2004). None of the studies point to climate change as a possible cause of the extinction of the Campo Miner.

### 4.2 Future predictions

Even with differences between models, it is possible to observe a marked reduction of suitable areas for all future scenarios, mainly in the states of Mato Grosso, Mato Grosso do Sul, Goiás, and Distrito Federal. This reduction is more dramatic in the pessimistic scenario, which projects a better suitability for the state of Minas Gerais. These results agree with other studies that evaluated the influence of climate and land use changes on bird communities in the Cerrado, which showed that the southern region of it will hold the largest refuge area for these species (Borges et al., 2019; Borges & Loyola, 2020; Marini et al., 2009a, 2009b). However, as acknowledged by Borges et al. (2019), the southern region is the most developed in the Cerrado, with much of its vegetation converted to pastures and crops (Sano et al., 2010), in addition to having the smallest conservation units (Françoso et al., 2015; Sano et al., 2019), which can be an impediment to the establishment of new bird populations or even to the conservation of species already existing in the region.

Even if the areas predicted by the model are indeed environmentally suitable for Campo Miners, other factors must also be considered. As a specialist grassland bird that tolerates only little changes in land use (Lopes et al., 2023; Machado et al., 2017; Meireles et al., 2018), the Campo Miner needs vast areas of natural grasslands, however, these areas are less common (Borges et al., 2019) and poorly represented in reserves. Protected areas are important because, by suffering less anthropogenic pressures and land use changes, they can, in addition to preserving species and environments, help to mitigate the effects of long‐term climate change (Dudley et al., 2009). Although Minas Gerais is the region with the best conditions for Campo Miners in the future, currently only one protected area (Serra da Canastra National Park) can be a stronghold for the preservation of the species in the state.

Some regions identified here proved to be extremely important for the creation of new protected areas, such as the Upper Rio Grande Grasslands (URRGs), which seems to be the most important stronghold for this resident species. However, the URGGS is poorly protected, with only two noteworthy reserves in the region, the Ibitipoca State Park (1488 ha) and the Wildlife State Refuge Libélulas da Serra de São José (3710 ha). These two small reserves, however, do not harbor suitable habitats for Campo Miners, which, ironically, is not protected within its main stronghold. The creation of large reserves in the URGGs should be a priority that will also benefit grassland specialists from other major taxonomic groups, including the Critically Endangered Leaf Frog *Pithecopus ayeaye* (Anura, Phyllomedusidae; Magalhães et al., 2017). The importance of this region is acknowledged since Drummond et al. (2005), who considered the mountains of São João‐del‐Rei and Tiradentes as of extreme priority for the creation of reserves. Another important region that deserves attention and needs further study is the Três Marias microregion, especially the municipality of Pompéu, which has a high number of records (http://www.wikiaves.com.br/mapaRegistros_andarilho) not only for Campo Miners but for several other threatened grassland birds (Souza et al., 2018). This region also does not have protected areas with suitable habitats to ensure the survival of the species.

The reserves would also protect a landscape that is naturally vulnerable and highly susceptible to erosive processes that lead to the loss of soil, habitat, and biodiversity (Lima et al., 2011). However, the strategy of creating new protected areas for the species needs a lot of caution, since, despite being a territorial species, Campo Miner shows strict habitat requirements, living in very open grasslands that are shaped by the fire regime and grazing pressure. Therefore, the habitat of the species needs to be carefully managed (Lopes et al., 2023).

The Campo Miner exhibits a suit of ecological requirements that can be very worrying for its conservation as it is a habitat specialist and territorial species (Lopes et al., 2021; Machado et al., 2017). Viana and Chase (2022), in their recent study on traits, ecology and demography of American birds and their relationship with climate change showed that habitat specialist and territorial species are more vulnerable to climate since they are more limited to specific environments and have a strong association with the type of vegetation. The impacts of climate, land use changes, and the consequent reduction in suitable areas can also have profound consequences on bird communities, as several other grassland species, including threatened ones, depend on the same habitat (Lopes & Peixoto, 2018; Lopes et al., 2010).

## 5 CONCLUSION

Campo Miner is a threatened species that has been suffering a marked reduction in its range in recent years and its survival in the face of global changes is not guaranteed. The effects of local extinctions are not only limited to the impact caused on its conservation, because interspecific interactions can be affected. Campo Miners build their nests within cavities they excavate in steep soil banks that are later used by other animals as shelter or even for nesting (Meireles et al., 2021), so its disappearance can cause impacts at the community level.

We show in this study a not optimistic future for the species since important areas with ideal environmental suitability will be considerably reduced. Even in an optimistic scenario of reduced greenhouse gas emissions and greater rigidity regarding land use policies, the species will lose a large part of its suitable areas. However, in a scenario of high emissions and high land degradation, the areas lost will be even greater, so an aggressive plan to mitigate CO_2_ emissions by limiting warming to 1.5°C by the end of the century as proposed (IPCC, 2022) and greater inspection of land use with effective policies and the creation of protected areas are very important to guarantee the future of the species.

Reducing CO_2_ emissions is crucial to ensure the future not only of the Campo Miner but also of several other bird species (Borges et al., 2019; Borges & Loyola, 2020; Marini et al., 2009a). However, as suggested by Marini et al. (2009a), plans are needed that assess not only climate change, but also land use change, as species distribution can be influenced by different factors. In addition, there is no point in areas that show good climatic suitability if they may have already been converted to pasture, agriculture, or even urbanized.

The state of Minas Gerais will shelter the largest area climatically suitable for the species in approximately 50 years. However, the lack of protected areas in the state with adequate habitats may be a hindrance to its preservation. As it is a nonmigratory bird (Lopes et al., 2023), it is likely that the main refuges for the species in the future will be within reserves, so the creation of new protected areas or even as mentioned by Lopes et al. (2023), a mosaic of fully protected and sustainable use reserves that cover their locations is very important to ensure the survival of Campo Miners. This is especially true in the Upper Rio Grande Grasslands, an area that is being silently devastated and that shelters the most important population of the species outside protected areas.

## AUTHOR CONTRIBUTIONS

**Ricardo C. Meireles:** Conceptualization (equal); data curation (equal); formal analysis (equal); methodology (equal); validation (equal); visualization (equal); writing – original draft (lead); writing – review and editing (equal). **Leonardo E. Lopes:** Conceptualization (equal); data curation (equal); supervision (supporting); writing – original draft (supporting); writing – review and editing (equal). **Gustavo R. Brito:** Conceptualization (equal); formal analysis (equal); methodology (equal); validation (equal); visualization (equal); writing – original draft (supporting); writing – review and editing (equal). **Ricardo Solar:** Conceptualization (equal); formal analysis (equal); funding acquisition (lead); methodology (equal); supervision (lead); writing – original draft (supporting); writing – review and editing (equal).

## CONFLICT OF INTEREST STATEMENT

No potential conflict of interest was reported by the authors.

## Supporting information


Appendix S1.

## Data Availability

All data used to generate this article are available at doi.org/10.5281/zenodo.7547448 and in public data platforms stated in the methods.

## References

[ece39802-bib-0001] Aiello‐Lammens, M. E. , Boria, R. A. , Radosavljevic, A. , Vilela, B. , & Anderson, R. P. (2015). spThin: An R package for spatial thinning of species occurrence records for use in ecological niche models. Ecography, 38(5), 541–545. 10.1111/ecog.01132

[ece39802-bib-0002] Alesp . (2018). Assembleia Legislativa do Estado de São Paulo. https://www.al.sp.gov.br/repositorio/legislacao/decreto/2018/decreto‐63853‐27.11.2018.html

[ece39802-bib-0003] Allouche, O. , Tsoar, A. , & Kadmon, R. (2006). Assessing the accuracy of species distribution models: Prevalence, kappa and true skill statistic (TSS). Journal of Applied Ecology, 43(6), 1223–1232. 10.1111/j.1365-2664.2006.01214.x

[ece39802-bib-0004] Álvares, C. A. , Stape, J. L. , Sentelhas, P. C. , de Moraes, G. J. L. , & Sparovek, G. (2013). Köppen's climate classification map for Brazil. Meteorologische Zeitschrift, 22(6), 711–728. 10.1127/0941-2948/2013/0507

[ece39802-bib-0005] Barbet‐Massin, M. , Jiguet, F. , Albert, C. H. , & Thuiller, W. (2012). Selecting pseudo‐absences for species distribution models: How, where and how many? Methods in Ecology and Evolution, 3(2), 327–338. 10.1111/j.2041-210X.2011.00172.x

[ece39802-bib-0006] Bates, J. M. , Parker, T. A., III , Caparella, A. , & Davis, T. J. (1992). Observations on the campo, cerrado and forest avifaunas of eastern Dpto. Santa Cruz, Bolivia, including 21 species new to the country. Bulletin of the British Ornithologists Club, 112(2), 86–98.

[ece39802-bib-0007] BirdLife International . (2022). Species factsheet: *Geositta poeciloptera* . http://datazone.birdlife.org/species/factsheet/campo‐miner‐geositta‐poeciloptera

[ece39802-bib-0008] BirdLife International and Handbook of the Birds of the World . (2021). Bird species distribution maps of the world. Version 2021.1. http://datazone.birdlife.org/species/requestdis

[ece39802-bib-0009] Borges, F. J. A. , & Loyola, R. (2020). Climate and land‐use change refugia for Brazilian Cerrado birds. Perspectives in Ecology and Conservation, 18(2), 109–115. 10.1016/j.pecon.2020.04.002

[ece39802-bib-0010] Borges, F. J. A. , Ribeiro, B. R. , Lopes, L. E. , & Loyola, R. (2019). Bird vulnerability to climate and land use changes in the Brazilian Cerrado. Biological Conservation, 236, 347–355. 10.1016/j.biocon.2019.05.055

[ece39802-bib-0011] Braz, V. S. (2008). Ecologia e conservação das aves campestres do bioma Cerrado. Tese para obtenção do título em Doutor em Ecologia. Universidade de Brasília, Brasília, Brasil.

[ece39802-bib-0012] Breiman, L. (2001). Random forests. Machine Learning, 45, 5–32. 10.1023/A:1010933404324

[ece39802-bib-0013] Bressan, P. M. , Kierulff, M. C. M. , & Sugieda, A. M. (2009). Fauna ameaçada de extinção no estado de São Paulo: vertebrados. São Paulo, Brazil. Fundação Parque Zoológico de São Paulo: Secretaria do Meio Ambiente, 648 p.

[ece39802-bib-0014] Busby, J. R. (1991). Bioclim, a bioclimatic analysis and prediction system. In C. R. Margules & M. P. Austin (Eds.), Nature conservation: Cost effective biological surveys and data analysis (pp. 64–68). CSIRO.

[ece39802-bib-0015] Dauby, G. (2020). ConR: Computation of Parameters Used in Preliminary Assessment of Conservation Status. R package version 1.3.0. https://CRAN.R‐project.org/package=ConR

[ece39802-bib-0016] del Castillo, H. , Clay, R. P. , Egea, J. , & Asociación Guyra Paraguay . (2005). Atlas de las aves de Paraguay. Asunción, Paraguay. Guyra Paraguay, 212 p.

[ece39802-bib-0017] Dornas, T. (2020). Registros relevantes e incrementos para a avifauna da região da Chapada dos Guimarães, bioma Cerrado, no Centro‐Oeste do Brasil. Atualidades Ornitológicas, 212, 1–8.

[ece39802-bib-0018] Drummond, G. M. , Martins, C. S. , Machado, Â. B. M. , Sebaio, F. A. , & Antonini, Y. (2005). Biodiversidade em Minas Gerais: Um atlas para sua conservação (2nd ed., 222 p.). Fundação Biodiversitas.

[ece39802-bib-0019] Dudley, N. , Stolton, S. , Belokurov, A. , Krueger, L. , Lopoukhine, N. , MacKinnon, K. , Sandwith, T. , & Sekhran, N. (2009). Natural Solutions: Protected Areas Helping People cope with climate change. IUCN‐WCPA, TNC, UNDP, WCS, the World Bank, and WWF, Gland, Switzerland, Washington, DC, and New York, USA, 130 p.

[ece39802-bib-0020] Elith, J. , Leathwick, J. R. , & Hastie, T. (2008). A working guide to boosted regression trees. Journal of Animal Ecology, 77(4), 802–813. 10.1111/j.1365-2656.2008.01390.x 18397250

[ece39802-bib-0021] ESRI . (2016). ArcGIS desktop: Release 10.5. Redlands, CA: Environmental Systems Research Institute.

[ece39802-bib-0022] Eyring, V. , Bony, S. , Meehl, G. A. , Senior, C. A. , Stevens, B. , Stouffer, R. J. , & Taylor, K. E. (2016). Overview of the coupled model Intercomparison project phase 6 (CMIP6) experimental design and organization. Geoscientific Model Development, 9(5), 1937–1958. 10.5194/gmd-9-1937-2016

[ece39802-bib-0023] Fick, S. E. , & Hijmans, R. J. (2017). WorldClim 2: New 1 km spatial resolution climate surfaces for global land areas. International Journal of Climatology, 37(12), 4302–4315. 10.1002/joc.5086

[ece39802-bib-0024] Fielding, A. H. , & Bell, J. F. (1997). A review of methods for the assessment of prediction errors in conservation presence/absence models. Environmental Conservation, 24(1), 38–49. 10.1017/S0376892997000088

[ece39802-bib-0025] Françoso, R. D. , Brandão, R. , Nogueira, C. C. , Salmona, Y. B. , Machado, R. B. , & Colli, G. R. (2015). Habitat loss and the effectiveness of protected areas in the Cerrado biodiversity hotspot. Natureza & Conservação, 13(1), 35–40. 10.1016/j.ncon.2015.04.001

[ece39802-bib-0026] Gibson, D. J. , & Newman, J. A. (2019). Grasslands and climate change: An overview. In D. Gibson & J. Newman (Eds.), Grasslands and climate change (ecological reviews) (pp. 3–18). Cambridge University Press.

[ece39802-bib-0027] Gidden, M. J. , Riahi, K. , Smith, S. J. , Fujimori, S. , Luderer, G. , Kriegler, E. , van Vuuren, D. P. , van den Berg, M. , Feng, L. , Klein, D. , Calvin, K. , Doelman, J. C. , Frank, S. , Fricko, O. , Harmsen, M. , Hasegawa, T. , Havlik, P. , Hilaire, J. , Hoesly, R. , … Takahashi, K. (2019). Global emissions pathways under different socioeconomic scenarios for use in CMIP6: A dataset of harmonized emissions trajectories through the end of the century. Geoscientific Model Development, 12(4), 1443–1475. 10.5194/gmd-12-1443-2019

[ece39802-bib-0028] Herzog, S. K. , Terrill, R. S. , Jahn, A. E. , Remsen, J. V. , Maillard, O. , García‐Soliz, V. H. , MacLeod, R. , Maccormik, A. , & Vidoz, J. Q. (2016). Birds of Bolivia: Field guide. Santa Cruz de la Sierra, Bolívia. Asociación Armonía, 496 p.

[ece39802-bib-0029] Hofmann, G. S. , Cardoso, M. F. , Alves, R. J. V. , Weber, E. J. , Barbosa, A. A. , Toledo, P. M. , Pontual, F. B. , Salles, L. O. , Hasenack, H. , Cordeiro, J. L. P. , Aquino, F. E. , & Oliveira, L. F. B. (2021). The Brazilian Cerrado is becoming hotter and drier. Global Change Biology, 27(17), 1–14. 10.1111/gcb.15712 34018296

[ece39802-bib-0030] ICMBio . (2011). Atlas da Fauna Brasileira Ameaçada de Extinção em Unidades de Conservação Federais. Brasília, Brazil, 276 p.

[ece39802-bib-0031] ICMBio . (2021a). Sumário executivo do plano de ação nacional para a conservação de aves do cerrado e pantanal. https://www.icmbio.gov.br/portal/faunabrasileira/plano‐de‐acao‐nacional‐lista/3618‐plano‐de‐acao‐nacional‐para‐a‐conservacao‐das‐aves‐do‐cerrado‐e‐pantanal

[ece39802-bib-0032] ICMBio . (2021b). Cerrado. https://www.icmbio.gov.br/portal/unidadesdeconservacao/biomas‐brasileiros/cerrado

[ece39802-bib-0033] IPCC . (2022). Climate change 2022: Impacts, adaptation, and vulnerability. In H.‐O. Pörtner , D. C. Roberts , M. Tignor , E. S. Poloczanska , K. Mintenbeck , A. Alegría , M. Craig , S. Langsdorf , S. Löschke , V. Möller , A. Okem , & B. Rama (Eds.), Contribution of working group II to the sixth assessment report of the intergovernmental panel on climate change. Cambridge University Press.

[ece39802-bib-0034] IUCN . (2012). IUCN Red List Categories and Criteria: Version 3.1 (2nd ed.). IUCN, Gland, Switzerland and Cambridge, UK. www.iucnredlist.org/technicaldocuments/categories‐and‐criteria

[ece39802-bib-0035] Köhler, M. , Esser, L. F. , Font, F. , Souza‐Chies, T. T. , & Majure, L. C. (2020). Beyond endemism, expanding conservation efforts: What can new distribution records reveal? Perspectives in Plant Ecology, Evolution and Systematics, 45, 125543. 10.1016/j.ppees.2020.125543

[ece39802-bib-0036] Li, X. , Chen, G. , Liu, X. , Liang, X. , Wang, S. , Chen, Y. , Pei, F. , & Xu, X. (2017). A new global land‐use and land‐cover change product at a 1‐km resolution for 2010 to 2100 based on human–environment interactions. Annals of the American Association of Geographers, 117(5), 1–20. 10.1080/24694452.2017.1303357

[ece39802-bib-0037] Lima, L. P. Z. , Louzada, J. , Carvalho, L. M. T. , & Scolforo, J. R. (2011). Análise da vulnerabilidade natural para a implantação de unidades de conservação na microrregião da Serra de Carrancas, MG. Cerne, 17(2), 151–159. 10.1590/S0104-77602011000200002

[ece39802-bib-0038] Liu, C. , Berry, P. M. , Dawson, T. P. , & Pearson, R. G. (2005). Selecting thresholds of occurrence in the prediction of species distributions. Ecography, 28(3), 385–393. 10.1111/j.0906-7590.2005.03957.x

[ece39802-bib-0039] Lopes, L. E. , Malacco, G. B. , Alteff, E. F. , Vasconcelos, M. F. , Hoffmann, D. , & Silveira, L. F. (2010). Range extensions and conservation of some threatened or little‐known Brazilian grassland birds. Bird Conservation International, 20(1), 84–94. 10.1017/S0959270909990190

[ece39802-bib-0040] Lopes, L. E. , Meireles, R. C. , Peixoto, H. J. C. , Teixeira, J. P. G. , Machado, T. L. S. S. , & Lombardi, V. T. (2023). Movement ecology of the threatened Campo Miner *Geositta poeciloptera* and its implications for conservation of tropical grassland birds. Bird Conservation International, 33, 1–11. 10.1017/S0959270922000417

[ece39802-bib-0041] Lopes, L. E. , & Peixoto, H. J. C. (2018). Aves campestres ameaçadas de extinção encontradas nos Campos do Alto Rio Grande, sul de Minas Gerais: notas sobre sua história natural e proposições para estudos futuros. Atualidades Ornitológicas, 201, 40–48.

[ece39802-bib-0042] Lopes, L. E. , Pinho, J. B. , Bernardon, B. , Oliveira, F. F. , Ferreira, L. P. , Vasconcelos, M. F. , Maldonado‐Coelho, M. , Nóbrega, P. F. A. , & Rubio, T. C. (2009). Aves da Chapada dos Guimarães, Mato Grosso, Brasil: Uma síntese histórica do conhecimento. Papéis Avulsos de Zoologia, 49(2), 9–47. 10.1590/S0031-10492009000200001

[ece39802-bib-0043] Lopes, L. E. , Teixeira, J. P. G. , Meireles, R. C. , Bastos, D. S. S. , Oliveira, L. L. , & Solar, R. (2021). High seasonal variation of plasma testosterone levels for a tropical grassland bird resembles patterns of temperate birds. Physiological and Biochemical Zoology, 94(3), 143–151. 10.1086/713503 33705275

[ece39802-bib-0044] Machado, T. L. S. S. , Lombardi, V. T. , Meireles, R. C. , Teixeira, J. P. G. , Solar, R. , & Lopes, L. E. (2017). Breeding biology of the threatened Campo Miner (*Geositta poeciloptera*) (Aves: Scleruridae), a Neotropical grassland specialist. Journal of Natural History, 51(41–42), 2551–2563. 10.1080/00222933.2017.1381771

[ece39802-bib-0045] Magalhães, R. F. , Lemes, P. , Camargo, A. , Oliveira, U. , Brandão, R. A. , Thomassen, H. , Garcia, P. C. A. , Leite, F. S. F. , & Santos, F. R. (2017). Evolutionarily significant units of the critically endangered leaf frog *Pithecopus ayeaye* (Anura, Phyllomedusidae) are not effectively preserved by the Brazilian protected areas network. Ecology and Evolution, 7(21), 8812–8828. 10.1002/ece3.3261 29177033 PMC5689491

[ece39802-bib-0046] Marini, M. Â. , Barbet‐Massin, M. , Lopes, L. E. , & Jiguet, F. (2009a). Predicted climate‐driven bird distribution changes and forecasted conservation conflicts in a neotropical savanna. Conservation Biology, 23(6), 1558–1567. 10.1111/j.1523-1739.2009.01258.x 19500118

[ece39802-bib-0047] Marini, M. Â. , Barbet‐Massin, M. , Lopes, L. E. , & Jiguet, F. (2009b). Major current and future gaps of Brazilian reserves to protect neotropical savanna birds. Biological Conservation, 142(12), 3039–3050. 10.1016/j.biocon.2009.08.002

[ece39802-bib-0048] McCullagh, P. , & Nelder, J. A. (1989). Generalized linear models (p. 532). Chapman and Hall/CRC.

[ece39802-bib-0049] Meireles, R. C. , Lopes, L. E. , Pichorim, M. , Machado, T. L. S. S. , Duca, C. , & Solar, R. (2021). Nest survival of the threatened Campo Miner *Geositta poeciloptera*: A tropical cavity‐nesting grassland bird. Austral Ecology, 46(8), 1236–1245. 10.1111/aec.13079

[ece39802-bib-0050] Meireles, R. C. , Teixeira, J. P. G. , Solar, R. , Vasconcelos, B. N. F. , Fernandes, R. B. A. , & Lopes, L. E. (2018). Soil attributes drive nest‐site selection by the Campo Miner *Geositta poeciloptera* . PLoS One, 13, e0192185. 10.1371/journal.pone.0192185 29381768 PMC5790285

[ece39802-bib-0051] MMA . (2022). Lista Nacional oficial de espécies da fauna ameaçadas de extinção. Ministério do Meio Ambiente. Diário Oficial da União – Portaria MMA nº 148, DE 7 DE JUNHO DE 2022.

[ece39802-bib-0052] Moraes, K. F. , Santos, M. P. D. , Gonçalves, G. S. R. , Oliveira, G. L. , Gomes, L. B. , & Lima, M. G. M. (2021). Climate change and bird extinctions in the Amazon. PLoS One, 16(5), e0252260. 10.1371/journal.pone.0236103 34015040 PMC8136666

[ece39802-bib-0053] Motta‐Junior, J. C. , Granzinolli, M. A. M. , & Develey, P. F. (2008). Aves da estação ecológica de Itirapina, estado de São Paulo, Brasil. Biota Neotropica, 8(3), 207–227. 10.1590/S1676-06032008000300019

[ece39802-bib-0054] Myers, N. , Mittermeier, R. A. , Mittermeier, C. G. , Fonseca, G. A. B. , & Kent, J. (2000). Biodiversity hotspots for conservation priorities. Nature, 403, 853–858. 10.1038/35002501 10706275

[ece39802-bib-0055] Naimi, B. , Hamm, N. , Groen, T. A. , Skidmore, A. K. , & Toxopeus, A. G. (2014). Where is positional uncertainty a problem for species distribution modelling? Ecography, 37, 191–203. 10.1111/J.1600-0587.2013.00205.X

[ece39802-bib-0056] Nix, H. A. (1986). A biogeographic analysis of Australian elapid snakes. In R. Longmore (Ed.), Atlas of Australian elapid snakes, Australian Flora and Fauna series N^o^ 7 (pp. 4–15). Australian Government Publishing Service.

[ece39802-bib-0057] Ortega, G. , Arias, P. A. , Villegas, J. C. , Marquet, P. A. , & Nobre, P. (2021). Present‐day and future climate over central and South America according to CMIP5/CMIP6 models. International Journal of Climatology, 41(15), 6713–6735. 10.1002/joc.7221

[ece39802-bib-0058] Parrini, R. , Raposo, M. A. , Pacheco, J. F. , Carvalhães, A. M. P. , Júnior, T. A. M. , Fonseca, P. S. M. , & Minns, J. (1999). Birds of the Chapada Diamantina, Bahia, Brazil. Cotinga, 11, 86–95.

[ece39802-bib-0059] Phillips, S. J. , Anderson, R. P. , & Schapire, R. E. (2006). Maximum entropy modeling of species geographic distributions. Ecological Modelling, 190(3–4), 231–259. 10.1016/j.ecolmodel.2005.03.026

[ece39802-bib-0060] R Development Core Team . (2021). R: A language and environment for statistical computing. R Foundation for Statistical Computing, Vienna. https://www.R‐project.org

[ece39802-bib-0061] Raes, N. , & Aguirre‐Gutiérrez, J. (2018). A modeling framework to estimate and project species distributions in space and time. In C. Hoorn , A. Perrigo , & A. Antonelli (Eds.), Mountains, climate and biodiversity (pp. 309–320). Wiley, J. & Sons.

[ece39802-bib-0062] Riahi, K. , van Vuuren, D. P. , Kriegler, E. , Edmonds, J. , O’Neill, B. C. , Fujimori, S. , Bauer, N. , Calvin, K. , Dellink, R. , Fricko, O. , Lutz, W. , Popp, A. , Cuaresma, J. C. , Samir, K. C. , Leimbach, M. , Jiang, L. , Kram, T. , Rao, S. , Emmerling, J. , … Tavoni, M. (2017). The shared socioeconomic pathways and their energy, land use, and greenhouse gas emissions implications: An overview. Global Environmental Change, 42, 153–168. 10.1016/j.gloenvcha.2016.05.009

[ece39802-bib-0063] Ridgely, R. S. , & Tudor, G. (2009). Field guide to the songbirds of South America: The Passerines. University of Texas Press. 748 p.

[ece39802-bib-0064] Sala, O. E. , Chapin, F. S., III , Armesto, J. J. , Berlow, E. , Bloomfield, J. , Dirzo, R. , Huber‐Sanwald, E. , Huenneke, L. F. , Jackson, R. B. , Kinzig, A. , Leemans, R. , Lodge, D. M. , Mooney, H. A. , Oesterheld, M. , Poff, N. L. , Sykes, M. T. , Walker, B. H. , Walker, M. , & Wall, D. H. (2000). Global biodiversity scenarios for the year 2100. Science, 287(5459), 1770–1774. 10.1126/science.287.5459.1770 10710299

[ece39802-bib-0065] Sano, E. E. , Rosa, R. , Brito, J. L. S. , & Ferreira, L. G., Jr. (2010). Land cover mapping of the tropical savanna region in Brazil. Environmental Monitoring and Assessment, 166, 113–124. 10.1007/s10661-009-0988-4 19504057

[ece39802-bib-0066] Sano, E. E. , Rosa, R. , Scaramuzza, C. A. M. , Adami, M. , Bolfe, E. L. , Coutinho, A. C. , Esquerdo, J. C. D. M. , Maurano, L. E. P. , Narvaes, I. S. , Filho, F. J. B. O. , Silva, E. B. , Victoria, D. C. , Ferreira, L. G. , Brito, J. L. S. , Bayma, A. P. , Oliveira, G. H. , & Bayma‐Silva, G. (2019). Land use dynamics in the Brazilian Cerrado in the period from 2002 to 2013. Pesquisa Agropecuária Brasileira, 54, e00138. 10.1590/S1678-3921.pab2019.v54.00138

[ece39802-bib-0067] Silveira, F. A. O. , Ordóñez‐Parra, C. A. , Moura, L. C. , Schmidt, I. B. , Andersen, A. N. , Bond, W. , Buisson, E. , Durigan, G. , Fidelis, A. , Oliveira, R. S. , Parr, C. , Rowland, L. , Veldman, J. W. , & Pennington, R. T. (2021). Biome awareness disparity is BAD for tropical ecosystem conservation and restoration. Journal of Applied Ecology, 59, 1967–1975. 10.1111/1365-2664.14060

[ece39802-bib-0068] Silveira, L. F. (2009). *Geositta poeciloptera* (Wied, 1830) Passeriformes, Scleruridae. In P. M. Bressan , M. C. M. Kierulff , & A. M. Sugieda (Eds.), Fauna ameaçada de extinção no estado de São Paulo: vertebrados. São Paulo, Brazil. Fundação Parque Zoológico de São Paulo: Secretaria do Meio Ambiente, pp. 201.

[ece39802-bib-0069] Souza, T. O. , Teixeira, F. D. , Oliveira, L. A. S. , Oliveira, A. C. S. , Garcia, F. I. A. , Mesquita, E. P. , Silva, G. G. R. , Oliveira, A. P. M. , Passos, M. F. O. , & Silva, A. G. (2018). Levantamento ornitológico do município de Pompéu, região Central de Minas Gerais, Brasil. Atualidades Ornitológicas, 202, 49–66.

[ece39802-bib-0070] Thuiller, W. , Georges, D. , Gueguen, M. , Engler, R. , & Breiner, F. (2021). Biomod2: Ensemble platform for species distribution modeling. R package version 3.5.1. https://cran.r‐project.org/package=biomod2

[ece39802-bib-0071] Velazco, S. J. E. , Villalobos, F. , Galvão, F. , & Júnior, P. M. (2019). A dark scenario for Cerrado plant species: Effects of future climate, land use and protected areas ineffectiveness. Diversity and Distributions, 25(4), 660–673. 10.1111/ddi.12886

[ece39802-bib-0072] Venables, W. N. , & Ripley, B. D. (2002). Modern applied statistics with S‐PLUS (4th ed.). Springer, 498 p.

[ece39802-bib-0073] Viana, D. S. , & Chase, J. M. (2022). Ecological traits underlying interspecific variation in climate matching of birds. Global Ecology and Biogeography, 31(5), 1021–1034. 10.1111/geb.13480

[ece39802-bib-0074] Willis, E. O. (2004). Birds of a habitat spectrum in the Itirapina savanna, São Paulo, Brazil (1982–2003). Brazilian Journal of Biology, 64(4), 901–910. 10.1590/S1519-69842004000500022 15744433

