# Supplementary material for: The future of suitable habitats of an endangered Neotropical grassland bird: A path to extinction?
Source: Ecol Evol. 2023 Feb 14;13(2):e9802. doi: 10.1002/ece3.9802 (PMC9926175; doi:10.1002/ece3.9802)
Supplement: Supplementary file 1 — Appendix S1. [file ECE3-13-e9802-s001.docx]

**Appendix**

**
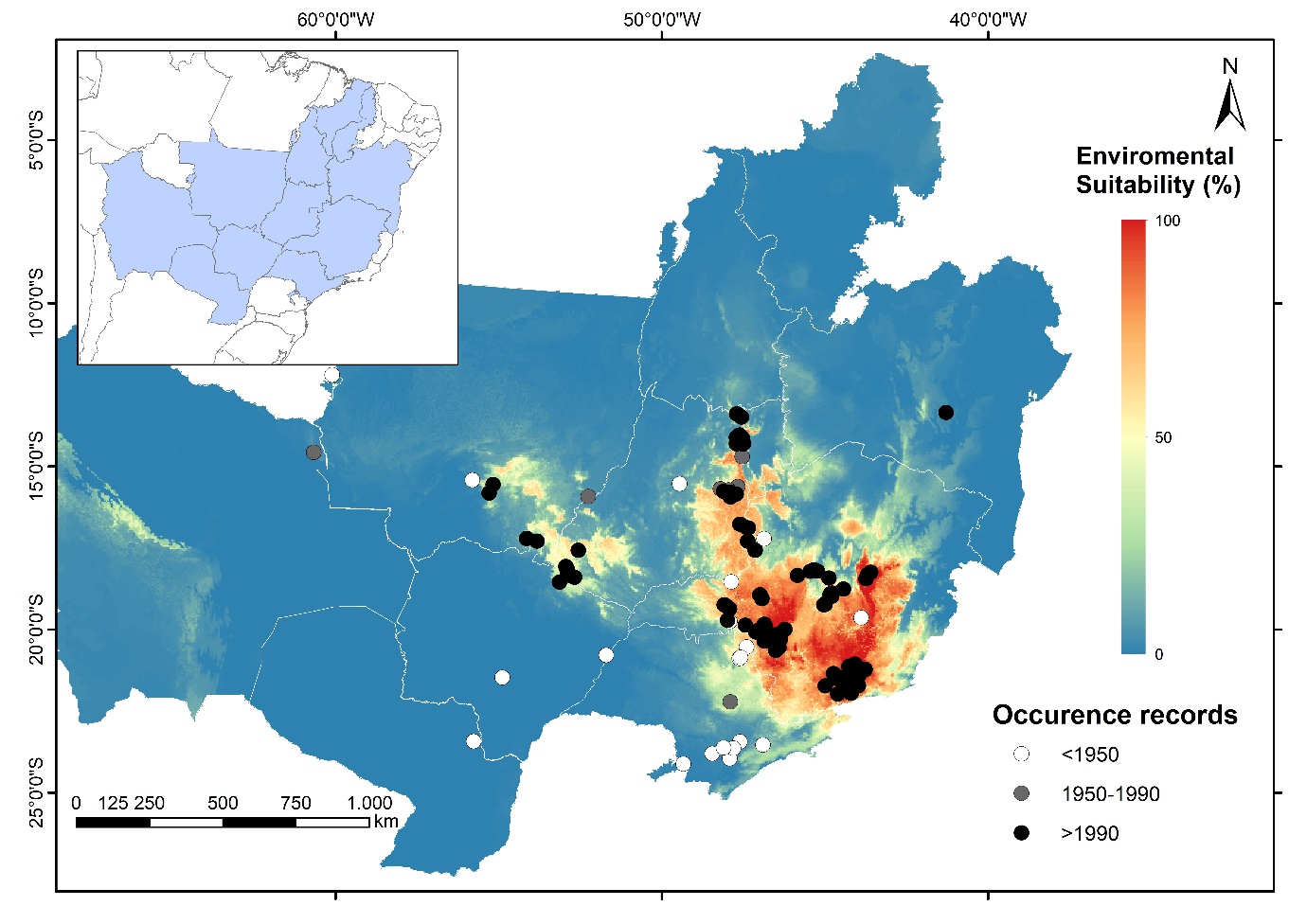
**

**Figure 1.** Current prediction and all occurrence records for Campo Miner.

**
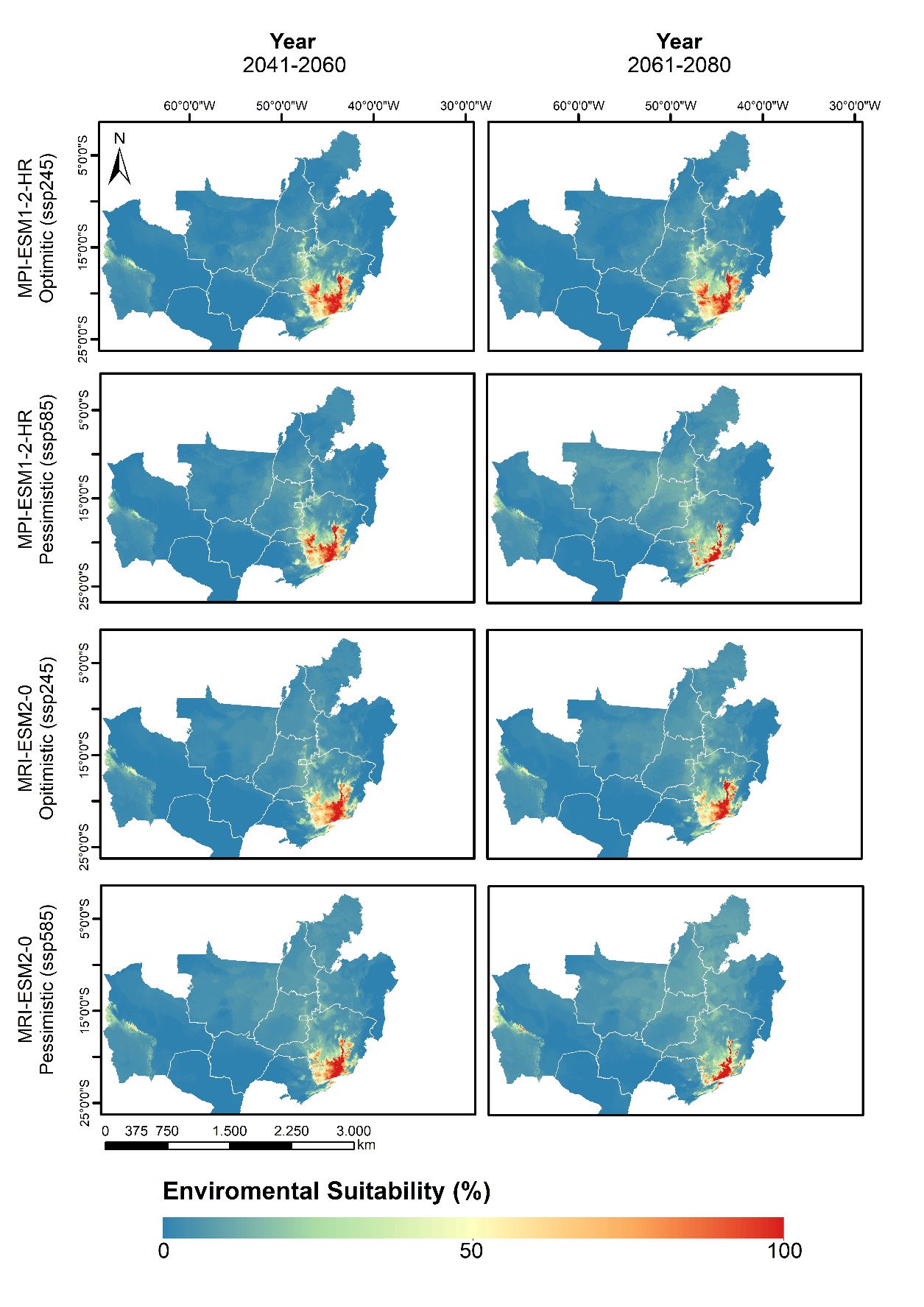
**

**Figure 2.** Future predictions of suitable areas for the occurrence of Campo Miner in Brazil in two different greenhouse gas emissions scenarios (ssp245 and ssp585) and GCMs (MPI-ESM1-2-HR and MRI-ESM2-0). Land use maps are related to the respective climate scenario (A1B for ssp245 and A2 for ssp585).
